# Long-term effects of cemented and cementless fixations of total knee arthroplasty: a meta-analysis and systematic review of randomized controlled trials

**DOI:** 10.1186/s13018-021-02762-2

**Published:** 2021-10-12

**Authors:** Cheng Chen, Yanyan Shi, Zhanpo Wu, Zengxin Gao, Youmin Chen, Changzheng Guo, Xianguo Bao

**Affiliations:** 1 Department of Orthopedics, Nanjing Lishui People’s Hospital, Zhongda Hospital Lishui Branch, Southeast University, No. 86 Chongwen Road, Lishui District, Nanjing, 211200 China; 2 Department of Geriatrics, Kong Jiang Hospital of Yangpu District, Shanghai, China

**Keywords:** Total knee arthroplasty, Cemented fixation, Prosthesis, Point motion

## Abstract

**Background:**

To determine the long-term effects (a minimum follow-up time 8.8 years) of cemented and cementless fixations used for total knee arthroplasty (TKA).

**Methods:**

PubMed, EMBASE, Ovid, Cochrane Library, CINAHL, China National Knowledge Infrastructure and China Wangfang database were interrogated for appropriate randomized controlled trials (RCTs) through July 2020. Data were extracted and assessed for accuracy by 2 of the authors acting independently. Any controversial discrepancies were resolved after discussion with a third author.

**Result:**

Eight RCTs were included with low to moderate bias risks. The cemented fixation of TKA was comparable to cementless fixation in terms of implant survival (relative risk, 1.016; 95% CI 0.978 to 1.056; *P* = 0.417), Knee Society (KS) knee score (standardized mean difference (SMD), − 0.107; 95% CI − 0.259 to 0.045; *P* = 0.168), KS function score (SMD − 0.065; 95% CI − 0.238 to 0.109; *P* = 0.463), KS pain score (SMD − 0.300; 95% CI − 0.641 to 0.042; *P* = 0.085), Western Ontario and McMaster Universities Osteoarthritis Index (WOMAC) score (SMD − 0.117; 95% CI − 0.307 to 0.073; *P* = 0.227), HSS score (SMD − 0.027; 95% CI − 0.270 to 0.217; *P* = 0.829), range of motion (SMD 0.061; 95% CI − 0.205 to 0.327; *P* = 0.652) at ≥ 8.8 years of follow-up. In terms of radiographic outcomes at ≥ 8.8 years of follow-up, the incidence of a radiolucent line in the cementless group was lower than for the cemented group (SMD 3.828; 95% CI 2.228 to 6.576; *P* < 0.001). However, the maximum total point motion (MTPM) of the cementless group was greater than for the cemented group (SMD − 0.739; 95% CI − 1.474 to − 0.005; *P* = 0.048).

**Conclusions:**

Long-term follow-up verified that cementless and cemented fixation have similar prosthesis survival rates, clinical scores and mobility. However, radiography suggested that each technique had an advantage with regard to the radiolucent line and MTPM.

**Supplementary Information:**

The online version contains supplementary material available at 10.1186/s13018-021-02762-2.

## Background

At present, total knee arthroplasty (TKA) is the recognized effective treatment for knee osteoarthritis (OA) and rheumatoid arthritis (RA). TKA can effectively relieve pain and improve knee function, thus improving the patient's a quality of life [1]. The fixation of a knee prosthesis is either cemented or non-cemented. It has been reported that 95.2% of total knee replacements have used cement for prosthesis fixation [2], and the application of cement prosthesis in clinical practice is considered to be the gold standard for TKA prosthesis fixation [3]. However, due to certain risks, such as mechanical trauma stimulation of foreign bodies during cement filling, fatal pulmonary embolism caused by the entry of small molecules of cement into the blood, and early loosening of the interface between bone and cement, there is grave concern about the long-term durability of cemented fixations. It has been suggested that a cementless prosthesis is used to replace the original cement fixation [4].

In recent years, the design concept and materials used to fabricate a cementless prosthesis have been greatly improved. Bioactive hydroxyapatite (HA) is attached to the prosthesis as a novel coating, increasing the contact surface between the implant and bone and becoming an attachment point for cells and fibrous membrane growth, which is conducive to bone growth in and around the prosthesis implant [5]. In terms of prosthetic metal materials, the trabecular metal prosthesis, which has the same elastic modulus as human bone tissue, features a multi-pore design to provide a more suitable biological environment for bone tissue growth [6]. Through stereo imaging analysis, it has been found that the new cementless prosthesis is superior to the traditional press fit design and cemented prostheses in reducing micro-motion, and it has been predicted that the risk of aseptic loosening will be greatly reduced [7].

The two fixation methods have their own advantages and disadvantages. The one which produces the longest prosthesis survival rate and better functional recovery is of great interest to clinical investigators. Currently, the published meta-analysis is mainly aimed at comparing the short and median-term effects of the 2 fixation methods. A meta-analysis [8] including 7 randomized controlled trials (RCTs) and quasi-RCTs with an average follow-up time of 7.1 years (range 2 to 16.6 years) found that the survival rate and clinical efficacy of the prosthesis was similar to complete cementless and cemented fixation. Similar revision rates and knee function improvements were also observed between the two fixation types in a meta-analysis [9] including RCTs with an average follow-up time of 8.4 years (range 2.0 to 16.6 years). In young patients (≤ 65 years old), cementless TKA was more effective regarding radiological [10] and clinical outcomes [11] compared to cemented TKAs. A meta-analysis [12] of 17 observational studies of individuals that had revision TKA, with ≥ 24-months follow-ups, found that there was no significant difference in failure, reoperation, aseptic loosening and infection between the cemented and non-cemented groups.

With the cohort of patients undergoing TKA becoming younger, so the demand for a longer survival rate of prostheses has increased [13]. The question of cemented or cementless fixation has once again become a hot topic of debate. It is known that the survival rate of a prosthesis is the most important indicator when evaluating the quality of fixation and is greatly related to the follow-up time. Therefore, RCTs with long-term follow-up were analyzed to compare the long-term efficacy of the 2 fixation methods in terms of implant survival rate, clinical scores and radiographic indicators, so as to provide a useful reference for the selection of a TKA prosthesis.

## Materials and methods

This systematic review and meta-analysis were conducted in accordance with PRISMA guidelines.

### Literature retrieval strategy

The Cochrane Library, PubMed, EMBASE, Ovid, CINAHL, China National Knowledge Infrastructure and China Wanfang databases were electronically interrogated for appropriate RCT studies on cemented and cementless fixations of TKA from the initial establishment of the database through July 2020. At the same time, the references of the included literatures were reviewed to ensure the integrity of the retrieved literature. The search terms were: total knee arthroplasty, TKA, total knee replacement, TKR, knee arthroplasty, knee replacement, cemented, cement, uncemented, uncement, cementless, randomized, randomized controlled trial and RCT. The specific Medline retrieval strategy is illustrated in Additional file 1: Table S1.

**Table 1:** Characteristics of included RCTs (*n* = 8)

Study	Year	Country	Time period of patient inclusion	No. of participants (C/U)	No. of knees^a^ (C/U)	Mean age (range, years)	Mean follow-up time (years)	Main outcomes^b^
Parker	2001	Canada	1987.1–1988.12	48/52	31/36	66.7 (50.6–76)	12.8	1
Baker	2007	UK	1987.6–1997.2	219/177	151/118	70.5 (41–88)	8.8	1
Park	2011	Korea	1997.1–1998.2	53/53	50/50	58.4 (51–67)	13.6	1, 2, 3, 4, 5, 7, 8
Pijls	2012	Netherlands	1993–1998	48 (total)	24/24 + 20^c^	65.71 (NR)	11–16 (range)	2, 3, 6, 7, 8, 9
Choy	2014	Korea	2002.1–2004.10	67/65	86/82	67.8 (49–80)	9.5	1, 2, 3, 5, 6, 7, 8
Kim	2014	Korea	1995.1–1996.3	85/85	80/80	54.3 (49–55)	16.6	1, 2, 5, 7, 8
Henricson	2018	Sweden	2003–2004	22/19	18/16	55 (33–59)	10	2, 3, 4, 7, 9
Batailler	2020	France	2004–2005	65/65	59/59	72 (50–90)	13	1, 2, 3, 7, 8

1, Survivorship; 2, Knee Society knee score; 3, Knee Society function score; 4, Knee Society pain score; 5, Western Ontario and McMaster Universities Osteoarthritis Index (WOMAC) score; 6, HSS score; 7, Range of motion (in degree); 8, Incidence of radiolucent line; 9, maximum total point motion (MTPM)

C, cemented group; U, uncemented group; NR, not reported

^a^At the last follow-up

^b^Included in the meta-analysis

^c^Cemented/HA-coated/uncoated tibial components (24/24/20)

### Inclusion and exclusion criteria

*Participants*: Patients with OA or RA of the knee who underwent TKA for the first time, regardless of age, race or nationality.

*Interventions*: the cementless group of patients were treated with cementless fixations (cementless femur component or cementless tibia component or both), while the cemented group were treated with cemented fixations (cemented femur component or cemented tibia component or both).

*Outcomes*: (1) Clinical indicators and scores: long-term survival rate of prosthesis (with any reason for revision as endpoint); Knee Society (KS) knee scores; KS functional scores; KS pain scores; Western Ontario and McMaster Universities Osteoarthritis Index (WOMAC) scores; Hospital for Special Surgery (HSS) scores; range of motion (ROM); (2) Radiographic indicators: Incidence of radiolucent line; maximum total point motion (MTPM). Outcome indicators were measured at the last follow-up. The included studies report at least one of the above indicators.

*Study design:* RCTs published only in Chinese or English.

*Exclusion criteria*: (1) for repeated published literatures, only the most complete or latest study were included; (2) the original literature data were not complete and could not be analyzed; (3) obvious preoperative ligament dysfunction or knee extension device dysfunction; (4) knee joint neuromuscular disease; (5) non-RCT study.

### Screening of literature and extraction of data

Two authors independently carried out literature screening and data extraction strictly following the defined inclusion/exclusion criteria, and also cross-checked the literature data. If there was any difference in their point of view, one author was consulted to assist judgment until a consensus was reached. The literature screening process was divided into primary and full-text screening, respectively.

#### Preliminary screening

By reading the title and abstract of the article, a judgement was made of its relevance to our research question; if it was closely related to our research topic, full-text screening was carried out.

#### Full text screening

We carefully read the full text of the literature, extracted relevant information and included studies that met our inclusion criteria. The relevant information of the literature was uniformly extracted into an Excel spreadsheet. The extracted information mainly included: (1) Basic information of the included research; title; first author name; year of publication; email address of the corresponding author; etc. (2) Information on the prosthesis used in each study: manufacturer; patella was resurfaced or not; posterior cruciate ligament (PCL)—retaining (Yes/No); the tibial component was fixed with or without screws; bearing type; etc. (3) Baseline information: patient age; gender; inclusion and exclusion criteria; intervention measures; etc. (4) Outcome information: follow-up time; number of knees followed; prosthesis survival rate; clinical scores; radiographic indicators; etc.

### Bias risk assessment of the included studies

Cheng Chen and Yanyan Shi independently evaluated the risk of bias in the included studies and cross-checked it. In case of disagreement, Zhanpo Wu was consulted to assist in the judgment until consensus was reached. Bias risk assessment software (Cochrane Collaboration Network) was employed to evaluate the bias risk of the RCTs including random methods, allocation concealment, blind implementation, complete data, selective reporting and other potential biases.

### Statistical analysis

Stata 13.0 software was used for all data analysis. The standardized mean difference (SMD) and the relative risk (RR) were used as effect analysis statistics (95% CI) for continuous variables and binary variables, respectively. A *χ*^2^ test was employed to analyze heterogeneity (test level set to *α* = 0.1), and the *I*^2^ was used to quantitatively judge the heterogeneity. If *I*^2^ was < 50 (*P* < 0.1) a fixed effect model was employed and if *I*^2^ was > 50 (*P* > 0.1) a random effect model was used. Analysis of subgroups for sensitivity analysis was employed to address any obvious clinical heterogeneity. A *P*-value < 0.05 was deemed to be a significant difference.

## Results

### Flow of the study

**Fig. 1:**
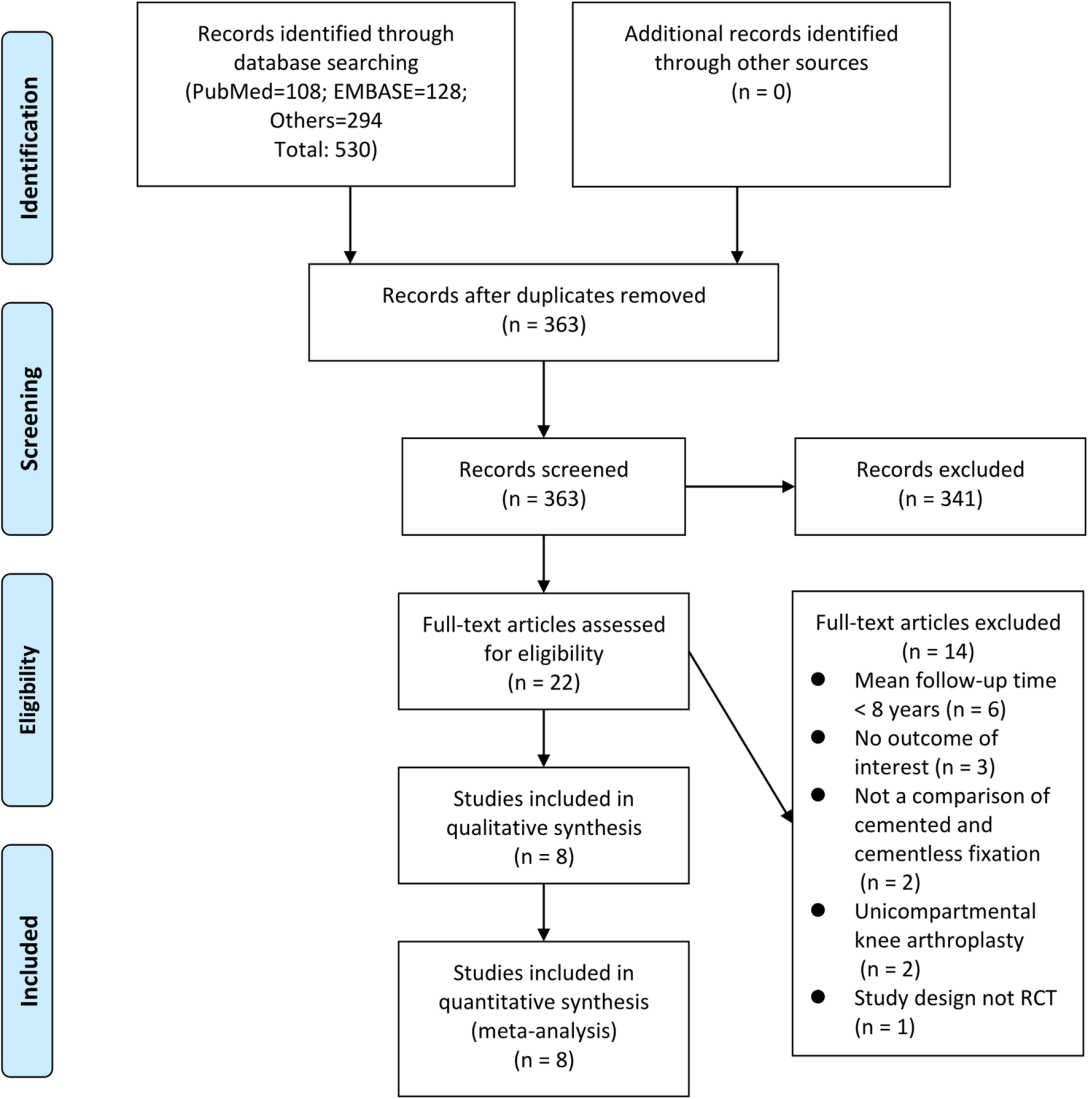
PRISMA flow diagram of the study selection

530 articles were retrieved from the searched databases (vide supra) using keywords related to cemented or cementless fixations of TKA, of which 167 duplicates were deleted. The title and abstract of each retrieved paper were carefully examined and those that were not related to the study topic deleted, with 22 articles remaining for full text analysis [1–8, 10, 12–24]. Six articles were excluded because the mean follow-up time was < 8 years, 3 study without outcomes of interest, 2 studies that were not comparisons of cemented and cementless fixation, 2 studies that investigated patients receiving unicompartmental knee arthroplasty and 1 study whose design was not an RCT. Finally, a total of 8 RCTs were included in our analysis (Fig. 1).

### Characteristics of the RCTs analyzed

1,123 patients (age range 41–90 years) were enrolled from 8 RCTs [25–32] conducted between 2001 and 2020. After over 8.8 years of follow-up (range 8.8–16.6 years), 984 knees at the last follow-up were included. We synthesized the evidence and assessed the long-term survivorship, clinical outcomes (KS knee scores, KS function scores, KS pain scores, WOMAC scores, HSS scores, ROM (degree)) and radiographic outcomes (incidence of radiolucent line, MTPM) by meta-analyses. Table 1 lists the main characteristics of patients in the included RCTs.

### Detailed information of total knee prostheses

A NexGen (Zimmer, Warsaw, US) prosthesis was used in 4 RCTs, a P.F.C. Sigma (Depuy, Warsaw, US) prosthesis in 2 RCTs, 1 RCT used an Interax (Howmedica, Rutherford, US) prosthesis and 1 study an HLS Noetos (Tornier, St-Ismier, France) prosthesis. All prostheses were fabricated to retain the posterior cruciate ligament with a fixed tibial structure. Three studies were concerned with resurfacing of the patella. None of the studies used screws to assist fixation. Four studies compared full-cemented with full-cementless TKA, 2 studies compared uncemented with cemented tibial component (with uncemented femoral component in both groups), 1 study compared uncemented with cemented femoral component (with cemented tibial component in both groups), and 1 study compared cemented with uncemented tibial component without reporting the condition of femoral component (Table 2).

**Table 2:** Detailed information on total knee prostheses

Study	Year	Manufacturer of prosthesis	Patella resurfaced	PCL retaining	Screw	Bearing type	Femoral component		Tibial component	
							C	U	C	U
Parker	2001	NexGen, Zimmer, Warsaw, Indiana	Yes	Yes	No	Fixed-bearing	Uncemented		Cemented	Uncemented
Baker	2007	P.F.C. Sigma, DePuy, Warsaw, US	No	Yes	No	Fixed-bearing	Cemented	Uncemented	Cemented	Uncemented
Park	2011	NexGen, Zimmer, Warsaw, Indiana	Yes	Yes	No	Fixed-bearing	Cemented	Uncemented	Cemented	Uncemented
Pijls	2012	Interax, Howmedica, Rutherford, US	NR	Yes	No	Fixed-bearing	NR		Cemented	Uncemented
Choy	2014	P.F.C. Sigma, DePuy, Warsaw, US	No	Yes	No	Fixed-bearing	Uncemented		Cemented	Uncemented
Kim	2014	NexGen, Zimmer, Warsaw, Indiana	Yes	Yes	No	Fixed-bearing	Cemented	Uncemented	Cemented	Uncemented
Henricson	2018	NexGen, Zimmer, Warsaw, US	A patella component was not routinely used	Yes	No	Fixed-bearing	Cemented	Uncemented	Cemented	Uncemented
Batailler	2020	HLS Noetos, Tornier, St-Ismier, France	NR	Yes	No	Fixed-bearing	Cemented	Uncemented	Cemented	

C, cemented group; U, uncemented group; NR, not reported

#### Quality of the RCTs

**Fig. 2:**
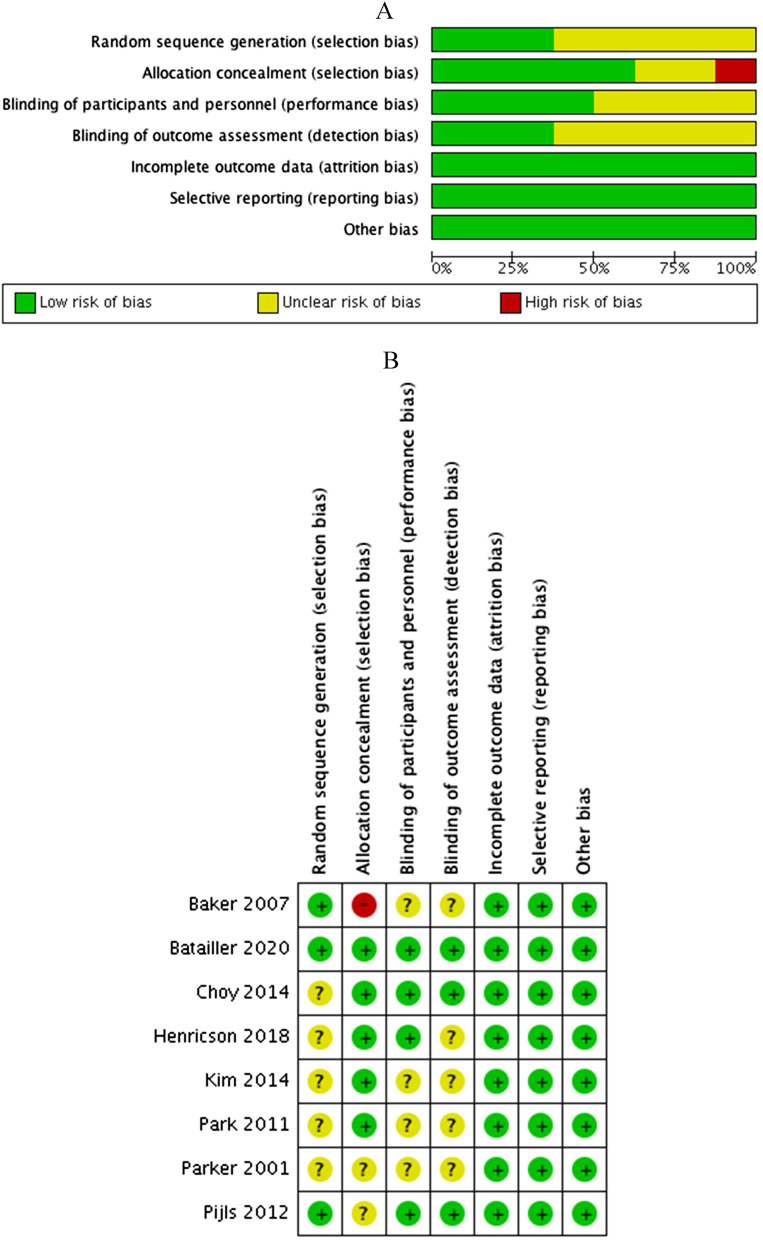
Risk of bias (**A**) graph and (**B**) summary showing the authors’ judgments about each risk of bias item presented as percentages across the 8 included RCTs

The bias risk plot and summary of the data are shown in Fig. 2A, B. Three of 8 studies analyzed random sequence generation, 5 allocation concealment, 4 blinding of patients and healthcare staff and 3 blinding of outcome assessments. All 8 studies had a low risk of attribution and reporting bias. Overall, the quality of the included studies varied from average to high quality.

#### Publication bias

The Egger test (Additional file 1: Figs. S1–S8) did not reveal any evidence for publication bias regarding implant survival (*P* = 0.140), KS knee scores (*P* = 0.130), KS function scores (*P* = 0.399), WOMAC scores (*P* = 0.908), HSS scores (*P* = 0.800), ROM (*P* = 0.704), the incidence of the radiolucent line (*P* = 0.051) or MTPM (*P* = 0.939). The test results of KS pain scores were not generally available because only 2 studies were included in the meta-analysis.

### Primary outcome

#### Implant survival

**Fig. 3:**
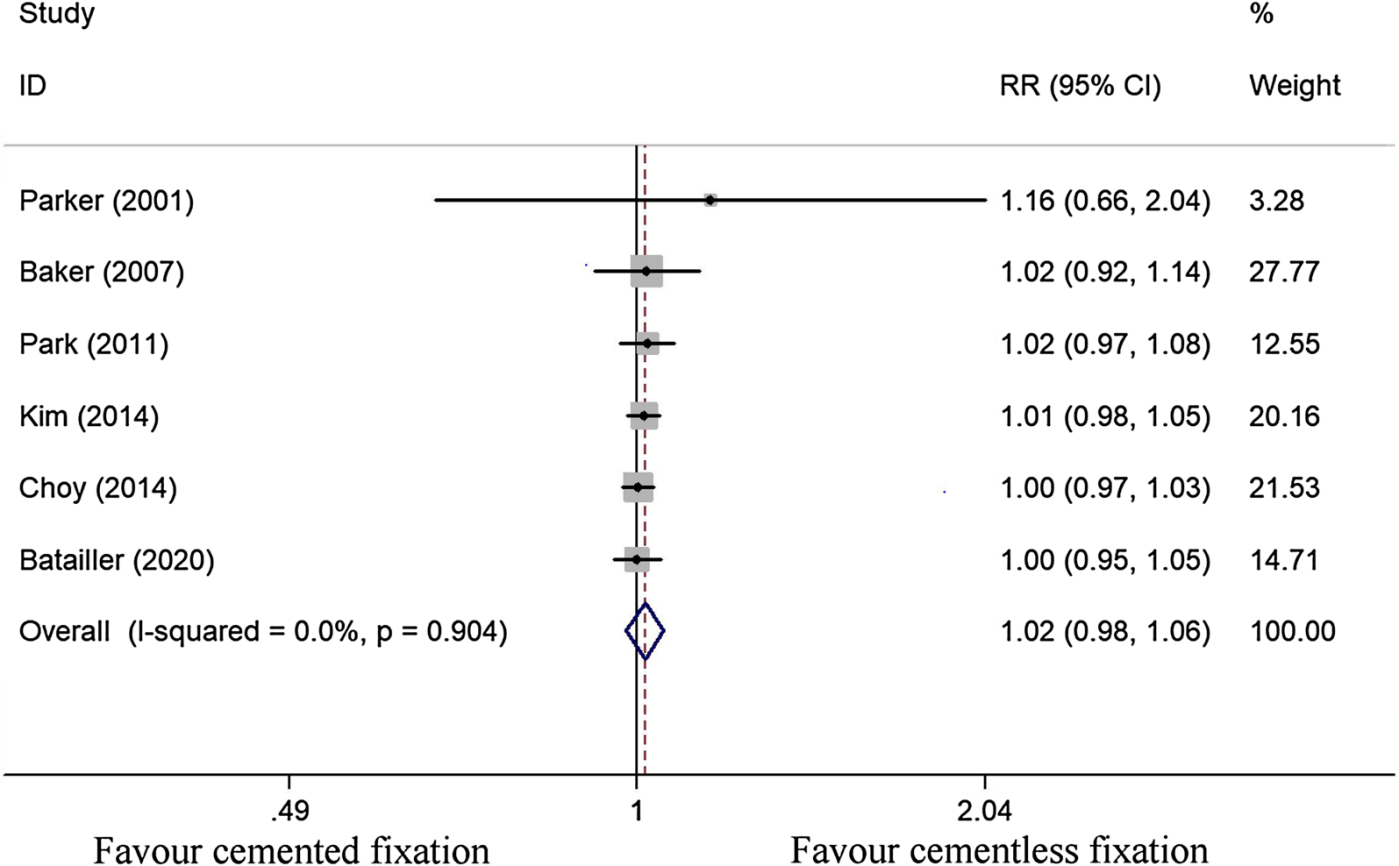
Comparison analysis of implant survival in the cemented and cementless fixation groups

We assessed the implant survival of cemented (*n* = 457) and cementless (*n* = 425) fixation of TKA by grouping together the survivorship at the last follow-up in 6 studies [25–27, 29, 30, 32]. After using a fixed effect model (*I*^2^ = 0%), the pooled data revealed the survivorship of the cementless fixation group of patients at a minimum of 8 years follow-up that was comparable to the cemented fixation group (RR, 1.016; 95% CI 0.978 to 1.056; *P* = 0.417) (Fig. 3).

### Secondary outcomes

#### Clinical outcomes

##### KS knee scores

**Fig. 4:**
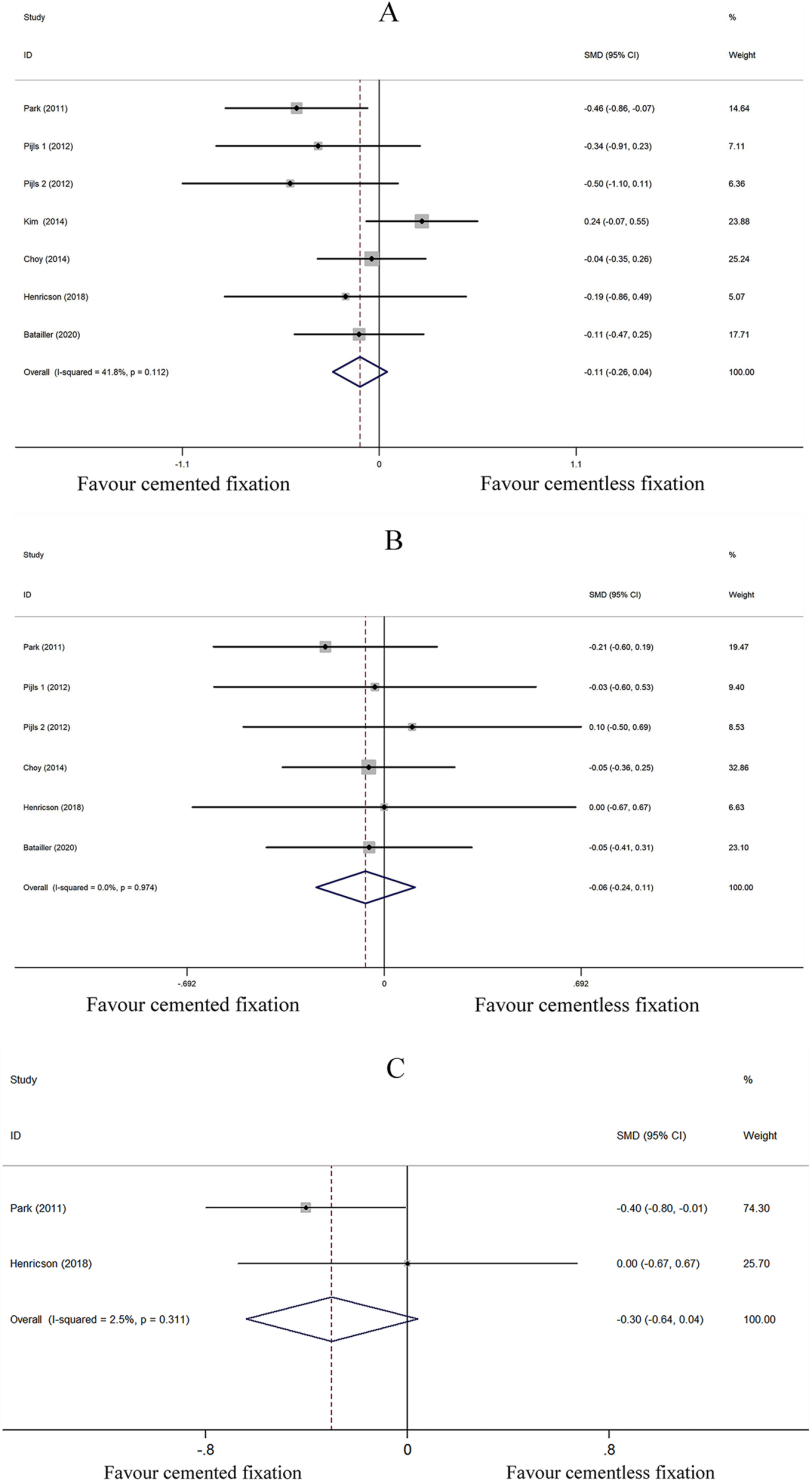
Comparison analysis of (**A**) Knee Society (KS) knee score (**B**) KS function score (**C**) KS pain score in the cemented and cementless fixation groups

We assessed the KS knee score of cemented (*n* = 298) and cementless (*n* = 317) fixation of TKA by grouping together the KS knee score at last follow-up in 6 studies [27–32]. After applying a fixed effect model (*I*^2^ = 41.8%), the pooled data indicated the KS knee score of cementless fixation group at minimum 8.8 years of follow-up is comparable to that of cemented fixation group (SMD − 0.107; 95% CI − 0.259 to 0.045; *P* = 0.168) (Fig. 4A).

##### KS function score

We assessed the KS function score of cemented (*n* = 218) and cementless (*n* = 237) fixation of TKA by grouping together the KS function score at last follow-up in 5 studies [27–29, 31, 32]. After employing the fixed effect model (*I*^2^ = 0%), pooled data analysis revealed that the KS knee scores of the cementless fixation group at a minimum 8.8 years follow-up were comparable to the cemented fixation group (SMD − 0.065; 95% CI − 0.238 to 0.109; *P* = 0.463) (Fig. 4B).

##### KS pain score

We assessed the KS pain score of cemented (*n* = 68) and cementless (*n* = 66) fixation of TKA by grouping together the KS pain score at last follow-up in 2 studies [27, 31]. After a fixed effect model (*I*^2^ = 2.5%) was applied, the pooled data revealed that the KS pain scores of the cementless fixation group of patients at ≥ 8.8 years of follow-up were comparable to those of the cemented fixation group (SMD − 0.300; 95% CI − 0.641 to 0.042; *P* = 0.085) (Fig. 4C).

##### WOMAC score

**Fig. 5:**
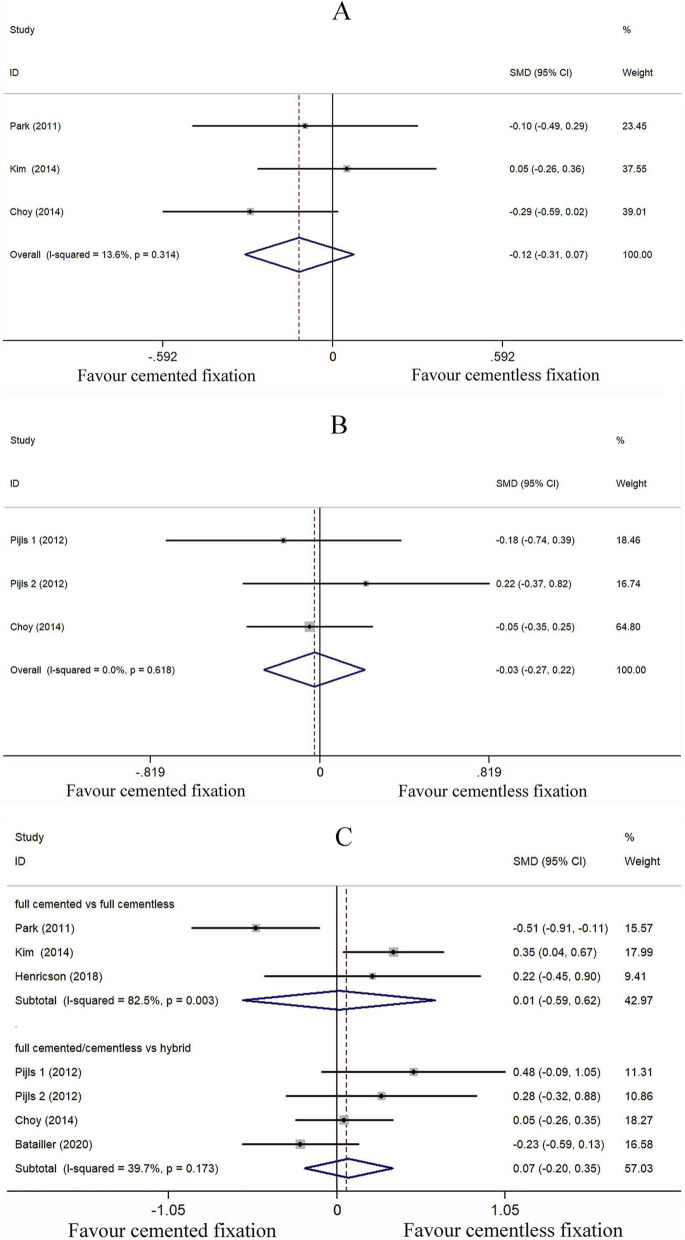
Comparison analysis of (**A**) WOMAC score (**B**) HSS score (**C**) range of motion in the cemented and cementless fixation groups

We assessed the WOMAC score of cemented (*n* = 216) and cementless (*n* = 212) fixation of TKA by grouping together the WOMAC score at last follow-up in 3 studies [27, 29, 30]. After the application of the fixed effect model (*I*^2^ = 13.6%), the pooled data revealed that the WOMAC scores of the cementless fixation group of patients at a minimum follow-up time of 8.8 years was comparable to the cemented fixation group (SMD − 0.117; 95% CI − 0.307 to 0.073; *P* = 0.227) (Fig. 5A).

##### HSS score

We assessed the HSS score of cemented (*n* = 110) and cementless (*n* = 126) fixation of TKA by grouping together the HSS score at last follow-up in 2 studies [28, 29]. After using the fixed effect model (*I*^2^ = 0%), the pooled data revealed that the HSS scores of the cementless fixation group at a minimum 8.8 years of follow-up were comparable to those of the cemented fixation group of patients (SMD − 0.027; 95% CI − 0.270 to 0.217; *P* = 0.829) (Fig. 5B).

###### ROM

We assessed the ROM (in degree) of cemented (*n* = 317) and cementless (*n* = 331) fixation of TKA by grouping together ROM at last follow-up in 6 studies [27–32]. Due to significant heterogeneity when using fixed model (*I*^2^ > 50%), we applied the random effect model. The pooled data revealed that the ROM of the cementless fixation group at minimum 8.8 years of follow-up was comparable to that of the cemented fixation group (SMD 0.061; 95% CI − 0.205 to 0.327; *P* = 0.652, *I*^2^ = 63.4%). We hypothesized that the inclusion of hybrid fixation might be a source of heterogeneity and did subgroup analysis of full cemented *vs* full cementless group and full cemented/cementless *vs* hybrid group. Similar results were also seen in the subgroup analysis (Fig. 5C).

### Radiographic outcomes

#### Incidence of radiolucent line

**Fig. 6:**
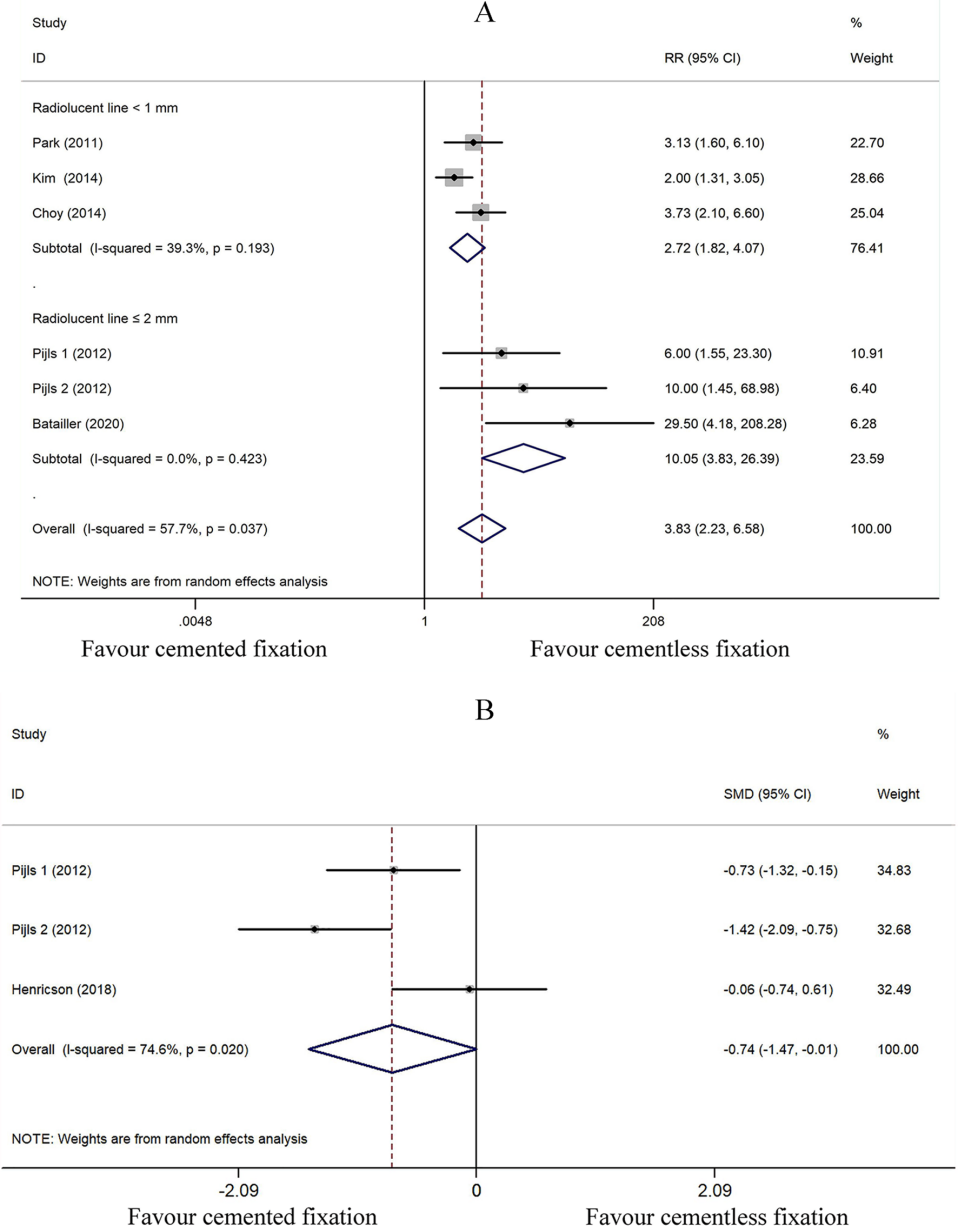
Comparison analysis of (**A**) incidence of radiolucent line (**B**) MTPM in the cemented and cementless fixation groups

We assessed the incidence of radiolucent line of cemented (*n* = 299) and cementless (*n* = 315) fixation of TKA by grouping together the incidence of radiolucent line at last follow-up in 5 studies [27–30, 32]. Due to significant heterogeneity (*I*^2^ = 57.7%), we applied the random effect model. Pooled data suggested that the incidence of the radiolucent line in the cementless fixation group of patients at a minimum of 8.8 years of follow-up was lower than that of the cemented fixation group (SMD 3.828; 95% CI 2.228 to 6.576; *P* < 0.001). We did a subgroup analysis according to the width of radiolucent line and found the heterogeneity greatly decreased. The incidence of radiolucent line < 1 mm in the cementless fixation group is significantly lower than that of the cemented fixation group (SMD 2.718; 95% CI 1.818 to 4.065; *P* < 0.001). A similar result was also observed in the subgroup of radiolucent line ≤ 2 mm (SMD 10.053; 95% CI 3.829 to 26.395; *P* < 0.001) (Fig. 6A).

#### MTPM

We assessed the MTPM (mm) of cemented (*n* = 42) and cementless (*n* = 60) fixation of TKA by grouping together the MTPM at last follow-up in 2 studies [28, 31]. Due to significant heterogeneity (*I*^2^ = 74.6%), the random effect model was employed. The pooled data strongly suggested that the MTPM of the cementless fixation group at a minimum 8.8 years follow-up was significantly greater than that of the cemented fixation group (SMD − 0.739; 95% CI − 1.474 to − 0.005; *P* = 0.048) (Fig. 6B).

## Discussion

A systematic review and meta-analysis of 8 RCT studies was carried out to investigate contemporary research results of the highest level of evidence for cemented or cementless fixation of TKAs. The available long-term evidence overwhelmingly indicated that cementless and cemented fixation have similar prosthesis survival rate, clinical scores, and mobility. However, cementless fixation causes less incidence of radiolucent line and more migration compared to cemented design.

Long term fixation is essential for cemented and cementless TKA, so the survival rate of prosthesis after TKA is one of the most important evaluation criteria in the clinical application of TKA [22, 24]. According to the results of previous studies, cemented fixation has advantages in early fixation and strength, while cementless fixation fails to show the same survival rate [14, 15]. In the meta-analysis published in 2009, Gandhi et al. [19] found that the failure rate of cementless fixation was higher by analyzing RCTs and observational studies. However, with the sophisticated development of prosthesis manufacturing technology, in contrast to previous research, recent reports [18, 21, 23] have shown that the survival rate of cementless prosthesis was similar to that of cemented prosthesis in short-term and medium-term follow-ups. Long-term follow-ups of TKA are essential before conclusions are made on the method of prosthesis fixation. Since most arthroplasty failures usually start near the 10-year mark [17], the significance of studies that have achieved good results in the short and medium-term follow-up is questionable in assessing prosthesis longevity. Therefore, it is necessary and worthwhile to obtain long-term follow-up data for a more complete understanding of both fixed conditions. In the 8 RCTs included in this meta-analysis, the minimum follow-up period was 8.8 years, and we found that the survival rate of cementless design was not inferior to the cemented design in the long-term follow-up. The possible reason is that the new cementless prosthesis design and improved prosthesis surface treatment increase the friction between prosthesis and bone tissue and allow better bone-implant biocompatibility, which is conducive to bone formation and bone growth, and can maintain long-term permanent fixation [20].

In our study, KS knee scores, KS function scores, WOMAC scores, HSS scores, and ROM were used to evaluate functional recovery after a TKA procedure. Our meta-analysis revealed that there were no significant differences between the 2 fixation groups at the last follow-up time. About 50% of TKA patients experienced moderate to severe postoperative pain [33], so pain control after TKA is an important clinical issue. The cementless group had no obvious advantage over the cemented group in terms of KS pain score. Due to the small heterogeneity of the included studies in clinical scores and the pooled analyses were based on relatively large group of 134 to 615 participants, it is reliable to conclude that similar good clinical and functional improvements were found in the two techniques after a minimum 8.8 years follow-up.

The presence and size of radiolucent lines as introduced by the Knee Society were described to assess the potential degree of osteolytic lesions. Radiolucent lines are likely associated with loosening or instability of the implant, including migration and inadequate load distribution. In the present study, the pooled analysis of the incidence of radiolucent lines, based on a large group of 614 patients, indicated that cementless fixation was linked with better long-term radiological outcomes compared to the cemented patient group. In the subgroup analysis, similar results were observed in both radiolucent line < 1 mm and radiolucent line < 2 mm groups with acceptable heterogeneity.

Radio stereometric analysis (RSA) offers an in vivo method of studying fixation. Five to 10 or more years of RSA-data may be more reliable to predict long-term fixation, since different types of fixation design may differ in their pattern of migration [28]. The pooled analysis of MTPM at minimum 8.8 years after operation demonstrated less component migration in the cemented group compared with cementless design. It is well acknowledged that cemented implants can initially achieve more stable fixation owing to its distinct migration patterns with cementless implants [34]. It seems that from a long-term perspective, cemented implants also show less migration. However, due to high heterogeneity (*I*^2^ = 74.6%), the result should be viewed with a degree of caution.

Despite the strict inclusion criteria of this study, there are still some limitations: First, half of the RCTs of hybrid fixation (not full cemented or cementless components) were included in our study, which may have introduced potential bias in selection. Second, over half of the studies didn’t report blinding of patients or outcome assessors, although blinding is often a problem in surgical trials [16], it may bias the meta-analysis. In addition, the identified language of only English and Chinese made publication bias inextricably.

## Conclusions

Similar survival rates and clinical performance were observed for both cemented and cementless TKAs at a minimum 8.8 years of follow-up. However, radiography suggests each has its advantage in radiolucent line and MTPM. It seems both designs could be acceptable options for primary TKA in the long-term perspective. In the future, it is urgent to conduct researches on the bone condition and demographic characteristics of patients to help surgeons select the most suitable fixation method for patients.

## Supplementary Information

**Additional file 1**. **Table S1**: MEDLINE search strategy. **Figure S1**: Egger's test survivorship. **Figure S2**: Egger's test KS knee score. **Figure S3**: Egger's test KS function score. **Figure S4**: Egger's test WAMAC score. **Figure S5**: Egger's test HSS score. **Figure S6**: Egger's test Range of motion. **Figure S7**: Egger's test radiolucent line. **Figure S8**: Egger's test MTPM.

## Data Availability

All data generated or analysed during this study are included in this published article and its Additional files.

## Abbreviations

HA: Hydroxyapatite
HSS: Hospital for special surgery
KS: Knee society
MTPM: Maximum total point motion
OA: Osteoarthritis
PCL: Posterior cruciate ligament
RA: Rheumatoid arthritis
RCT: Randomized controlled trials
ROM: Range of motion
RR: Relative risk
RSA: Radio stereometric analysis
SMD: Standardized mean difference
TKA: Total knee arthroplasty
WOMAC: Western Ontario and McMaster Universities Osteoarthritis Index
