# Supplementary material for: Long-term effects of cemented and cementless fixations of total knee arthroplasty: a meta-analysis and systematic review of randomized controlled trials
Source: J Orthop Surg Res. 2021 Oct 12;16:590. doi: 10.1186/s13018-021-02762-2 (PMC8513310; doi:10.1186/s13018-021-02762-2)
Supplement: Supplementary file 1 — Additional file 1. Table S1: MEDLINE search strategy. Figure S1: Egger's test survivorship. Figure S2: Egger's test KS knee score. Figure S3: Egger's test KS function score. Figure S4: Egger's test WAMAC score. Figure S5: Egger's test HSS score. Figure S6: Egger's test Range of motion. Figure S7: Egger's test radiolucent line. Figure S8: Egger's test MTPM. [file 13018_2021_2762_MOESM1_ESM.docx]

**Supplemental Table 1. MEDLINE search strategy**

|  | **MEDLINE search strategy** |
| --- | --- |
| #1 | ((arthroplasty, knee replacement [MeSH Terms]) OR (((knee arthroplasty [Title/Abstract]) OR (knee replacement [Title/Abstract])) OR (knee prosthesis [Title/Abstract]))) OR ((TKA [Title/Abstract]) OR (TKR [Title/Abstract])) |
| #2 | (cement [Title/Abstract]) OR (cemented [Title/Abstract]) |
| #3 | (uncemented [Title/Abstract]) OR (cementless [Title/Abstract]) |
| #4 | (((random*[Title/Abstract]) OR (randomized controlled trial [Publication Type])) OR (randomized controlled trials as topic [MeSH Terms])) OR (Randomized Controlled Trial [Title/Abstract]) |
| #5 | #1 AND #2 AND #3 AND #4 |


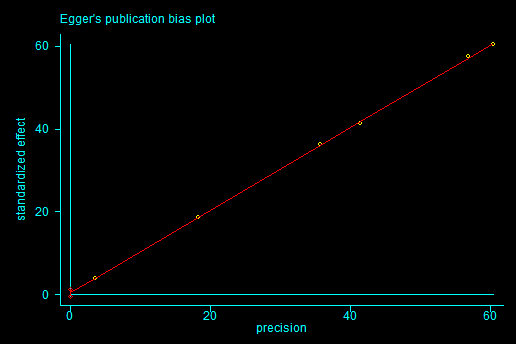


**Supplementary Figure 1. Egger's test survivorship**


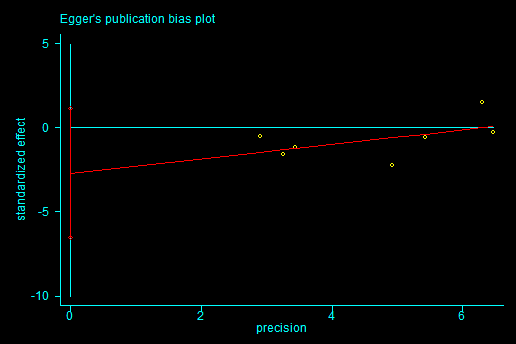


**Supplementary Figure 2. Egger's test KS knee score**


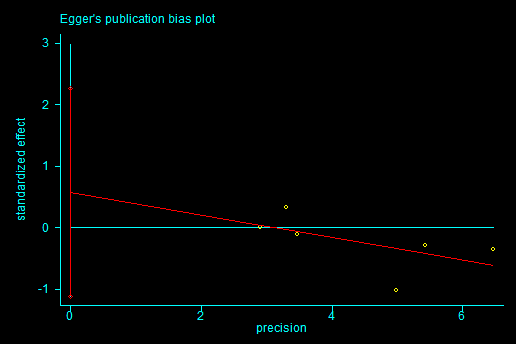


**Supplementary Figure 3. Egger's test KS function score**


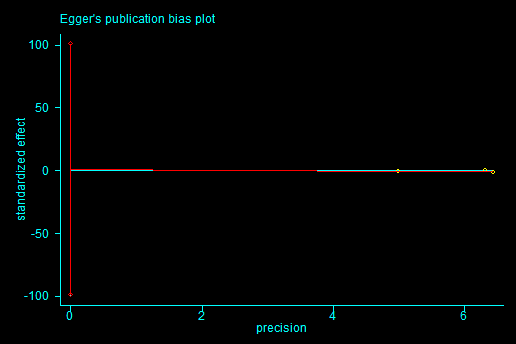


**Supplementary Figure 4. Egger's test WAMAC score**

**
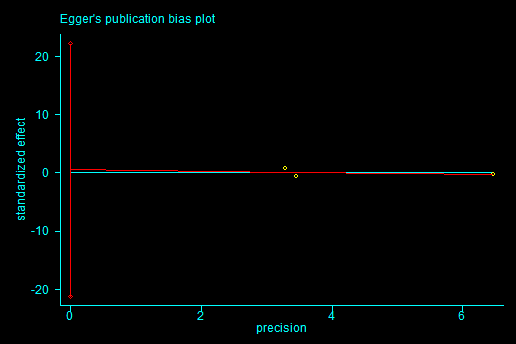
**

**Supplementary Figure 5. Egger's test HSS score**

**
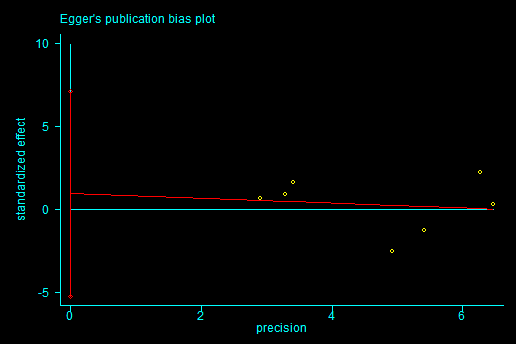
**

**Supplementary Figure 6. Egger's test Range of motion**

**
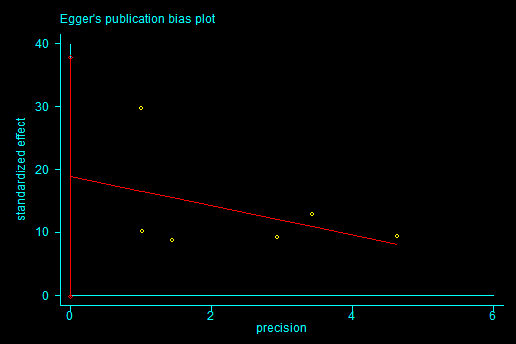
**

**Supplementary Figure 7. Egger's test radiolucent line**

**
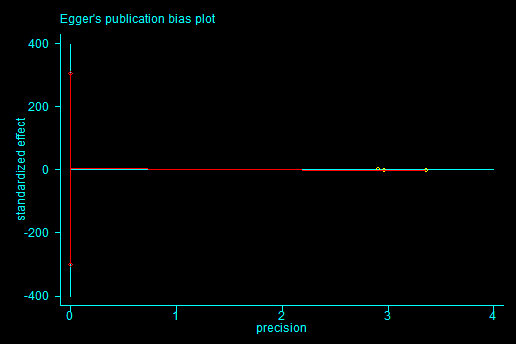
**

**Supplementary Figure 8. Egger's test MTPM**
